# New Insights into the Diversity of the Genus *Faecalibacterium*

**DOI:** 10.3389/fmicb.2017.01790

**Published:** 2017-09-22

**Authors:** Leandro Benevides, Sriti Burman, Rebeca Martin, Véronique Robert, Muriel Thomas, Sylvie Miquel, Florian Chain, Harry Sokol, Luis G. Bermudez-Humaran, Mark Morrison, Philippe Langella, Vasco A. Azevedo, Jean-Marc Chatel, Siomar Soares

**Affiliations:** ^1^Department of General Biology, Federal University of Minas Gerais Belo Horizonte, Brazil; ^2^Commensals and Probiotics-Host Interactions Laboratory, Micalis Institute, Institut National de la Recherche Agronomique, AgroParisTech, Université Paris-Saclay Jouy-en-Josas, France; ^3^Faculty of Medicine, Translational Research Institute, University of Queensland Diamantina Institute, University of Queensland St. Lucia, QLD, Australia; ^4^UMR 6023 Laboratoire Microorganismes: Génome et Environnement, Centre National de la Recherche Scientifique, Université Clermont Auvergne Clermont-Ferrand, France; ^5^Faculté de Médecine Saint-Antoine, Institut National de la Santé et de la Recherche Médicale U1157/UMR7203, AVENIR Team Gut Microbiota and Immunity Equipe de Recherche Labélisée, Université Pierre et Marie Curie Paris, France; ^6^Service de Gastroentérologie, Hôpital Saint-Antoine, Assistance Publique—Hôpitaux de Paris Paris, France; ^7^Department of Immunology, Microbiology and Parasitology, Institute of Biological Sciences and Natural Sciences, Federal University of Triângulo Mineiro Uberaba, Brazil

**Keywords:** *Faecalibacterium prausnitzii*, genome sequencing, 16S rRNA gene phylogeny, phylogenomic analysis, Average Nucleotide Identity, gene synteny, pangenome, new species

## Abstract

*Faecalibacterium prausnitzii* is a commensal bacterium, ubiquitous in the gastrointestinal tracts of animals and humans. This species is a functionally important member of the microbiota and studies suggest it has an impact on the physiology and health of the host. *F. prausnitzii* is the only identified species in the genus *Faecalibacterium*, but a recent study clustered strains of this species in two different phylogroups. Here, we propose the existence of distinct species in this genus through the use of comparative genomics. Briefly, we performed analyses of 16S rRNA gene phylogeny, phylogenomics, whole genome Multi-Locus Sequence Typing (wgMLST), Average Nucleotide Identity (ANI), gene synteny, and pangenome to better elucidate the phylogenetic relationships among strains of *Faecalibacterium*. For this, we used 12 newly sequenced, assembled, and curated genomes of *F. prausnitzii*, which were isolated from feces of healthy volunteers from France and Australia, and combined these with published data from 5 strains downloaded from public databases. The phylogenetic analysis of the 16S rRNA sequences, together with the wgMLST profiles and a phylogenomic tree based on comparisons of genome similarity, all supported the clustering of *Faecalibacterium* strains in different genospecies. Additionally, the global analysis of gene synteny among all strains showed a highly fragmented profile, whereas the intra-cluster analyses revealed larger and more conserved collinear blocks. Finally, ANI analysis substantiated the presence of three distinct clusters—A, B, and C—composed of five, four, and four strains, respectively. The pangenome analysis of each cluster corroborated the classification of these clusters into three distinct species, each containing less variability than that found within the global pangenome of all strains. Here, we propose that comparison of pangenome subsets and their associated α values may be used as an alternative approach, together with ANI, in the *in silico* classification of new species. Altogether, our results provide evidence not only for the reconsideration of the phylogenetic and genomic relatedness among strains currently assigned to *F. prausnitzii*, but also the need for lineage (strain-based) differentiation of this taxon to better define how specific members might be associated with positive or negative host interactions.

## Introduction

Members of genus *Faecalibacterium* are commensal bacteria, ubiquitous in the gastrointestinal tracts of animals and humans. Within the human colon, this taxon is the main member of the *Clostridium leptum* cluster, and comprises the second-most common representative in fecal samples, after *Clostridium coccoides* (Tap et al., 2009; Walker et al., 2011). The first characterized isolates were classified as *Fusobacterium prausnitzii*, but its close relationship with other members of the *C. leptum* cluster (phylum Firmicutes, class Clostridia, family Ruminococcaceae) was later established through analysis of the 16S rRNA gene of different strains found in humans (ATCC 27766 and ATCC 27768) (Wang et al., 1996; Duncan et al., 2002). The relative abundance of *F. prausnitzii* in vertebrate animals other than humans, such as pigs (Castillo et al., 2007), mice (Nava and Stappenbeck, 2011), calves (Oikonomou et al., 2013), and chickens (Scupham, 2007), suggests that the species is a functionally important member of the microbiota and likely has an impact on the physiology and health of the host. In that context, changes in the abundance of *F. prausnitzii* have been widely described in various intestinal and metabolic diseases in humans, such as colorectal cancer (CRC), Crohn's disease (CD), and ulcerative colitis (UC) (Sokol et al., 2008; Rajilić-Stojanović et al., 2011; Miquel et al., 2013). Due to its ubiquity and immunomodulatory properties, some studies suggest that *F. prausnitzii* is an indicator of, and an active contributor to, intestinal health and the maintenance of gut homeostasis (Sokol et al., 2008; Miquel et al., 2013, 2014). Despite its relevance in the human gut ecosystem, little is known about the diversity of *F. prausnitzii* (Miquel et al., 2014) and only a few studies have examined isolated strains and used functional approaches (Duncan et al., 2002; Lopez-Siles et al., 2012). To better understand the biodiversity and beneficial effect of this species, it is essential to increase our knowledge of several cultured strains and their genomes.

Recent studies, based on 16S rRNA sequence analyses, have suggested the existence of two phylogroups within this species (Duncan et al., 2002; Lopez-Siles et al., 2012, 2017). Here we present a new phylogenetic and comparative study of five sequenced genomes of *F. prausnitzii* available in public databases, combined with twelve new genome sequences isolated from healthy volunteers in Europe and Australia. The phylogenetic relationships among these isolates of *F. prausnitzii* were compared, and pangenomic analyses provided us a more global view of the genomic diversity across these strains. These data will enable new insights into the contributions of genus *Faecalibacterium* to gut function.

## Methods

### Genome sequencing, assembly, and annotation

The genomes used in this study are presented in Table 1. The genome data of five different *F. prausnitzii* strains were retrieved from the PATRIC public database. These were combined with genome data from ten newly isolated *F. prausnitzii* strains recovered from stool samples of healthy European volunteers (Martín et al., 2017), as well as two newly isolated *F. prausnitzii* strains recovered from stool samples of healthy Australian subjects (following the guidelines of the University of Queensland Human Research Ethics Committee #2015000775). In Europe, the ten new genomes were sequenced by GATC Biotech Company using the Illumina HiSeq2500 platform; the genomes from the Australian isolates were sequenced using the Illumina NextSeq platform at the Australian Centre for Ecogenomics (www.ecogenomic.org). The genome of the wild-type strain *F. prausnitzii* A2-165 (*F. prausnitzii*_A2-165_PacBio) was sequenced using PacBio single-molecule real-time (SMRT) technology on an RS system (Pacific Bioscience) and assembled by the GATC Biotech Company. The quality of the sequenced reads was checked with FastQC software (http://www.bioinformatics.babraham.ac.uk/projects/fastqc/). All of the genomes (except for wild-type strain A2-165) were assembled using a *de novo* strategy with SPAdes software, v3.8.0. The quality of the assemblies was evaluated using QUAST software (Gurevich et al., 2013) and all genomes were subjected to automated functional annotation using the RAST server (Aziz et al., 2008).

**Table 1:** Genomic features of *F. prausnitzii* genomes.

**Genome name**	**PATRIC genome ID**	**Genbank accessions**	**Isolation country**	**Sequences**	**Genome length**	**GC content**	**PATRIC CDS**
*Faecalibacterium prausnitzii M21/2*	411485.10	ABED00000000	United Kingdom	25	3,125,761	56.3	2,776
*Faecalibacterium prausnitzii SL3/3*	657322.3	FP929046	United Kingdom	1	3,214,418	54.81	3,052
*Faecalibacterium prausnitzii L2-6*	718252.3	FP929045	United Kingdom	1	3,321,367	55.57	3,232
*Faecalibacterium cF. prausnitzii KLE1255*	748224.3	AECU00000000	USA	139	2,907,000	56.27	2,783
*Faecalibacterium prausnitzii AHMP_21*	853.123	NOUV00000000	Australia	85	3,019,317	57.36	3,201
*Faecalibacterium prausnitzii HMI_19*	853.124	NOUW00000000	Australia	63	2,879,169	56.82	2,933
*Faecalibacterium prausnitzii CNCM_4540*	853.62	NMTQ00000000	France	48	3,043,568	55.7	3,206
*Faecalibacterium prausnitzii CNCM_4541*	853.63	NMTR00000000	France	78	2,822,838	58.11	2,825
*Faecalibacterium prausnitzii CNCM_4542*	853.64	NMTS00000000	France	106	2,914,466	55.83	3,071
*Faecalibacterium prausnitzii CNCM_4543*	853.65	NMTT00000000	France	22	3,080,452	56.2	3,223
*Faecalibacterium prausnitzii CNCM_4544*	853.66	NMTU00000000	France	71	2,808,526	55.98	2,907
*Faecalibacterium prausnitzii CNCM_4546*	853.67	NMTV00000000	France	244	3,422,520	54.88	3,611
*Faecalibacterium prausnitzii CNCM_4573*	853.68	NMTW00000000	France	83	3,275,249	55.9	3,479
*Faecalibacterium prausnitzii CNCM_4574*	853.69	NMTX00000000	France	38	3,088,985	56.26	3,249
*Faecalibacterium prausnitzii CNCM_4575*	853.70	NMTY00000000	France	37	3,006,602	57.51	3,077
*Faecalibacterium prausnitzii CNCM_4644*	853.71	NMTZ00000000	France	36	2,915,240	56.37	3,019
*Faecalibacterium prausnitzii A2-165_PacBio*	853.73	CP022479	France	1	3,110,044	56.33	3,231

### Phylogeny

Phylogenetic analyses were performed on 16S rRNA sequences, whole genome sequences, and the results of whole-genome Multi-Locus Sequence Typing (wgMLST). For the first analysis, the 16S rRNA sequence from the genome of *F. prausnitzii*_A2-165_PacBio was used to perform a BLASTn search in the NCBI database for all sequences belonging to the genus *Faecalibacterium*. The sequence results with more than 82% coverage and 92% identity were collected, and the 16S rRNA gene sequence from *Subdoligranulum variabile* BI-114 was included as an outgroup. Sequences were then aligned using the multiple sequence alignment tool CLUSTALW (Thompson et al., 1994) integrated in MEGA7 software (Kumar et al., 2016). After that, the most appropriate evolutionary model was defined and evolutionary history was inferred using the maximum-likelihood (ML) criterion, based on the Kimura 2-parameter model (Kimura, 1980), with 1,000 bootstrap replicates.

The phylogenomic analysis was performed using Gegenees software (Agren et al., 2012), which calculated the percentage of similarity among the genomes of all strains. Before calculating similarity scores, we used the BLASTn alignment method, with a sequence fragmentation length of 200 bp and a step size of 100 bp. The input files for Gegenees contained the complete genomes in “.fna” format and the resulting similarity matrix was exported in “.nexus” format for phylogenomic analysis using SplitsTree4 software (Huson, 2005). The equal angle method was used to construct the phylogenetic network, which was plotted with NeighborNet.

A wgMLST analysis was performed using the *Build_wgMLSTtree* module in the PGAdb-builder web service tool (Liu et al., 2016). The 17 genome sequences were compared with the resulting PGAdb profile using BLASTn, with filters of 80% coverage and 90% identity.

### Average nucleotide identity

We also performed an Average Nucleotide Identity (ANI) analysis using the whole-genome sequences. ANI represents a mean of identity/similarity values between homologous genomic regions shared by two genomes. It is generally accepted that ANI values of 95–96% equate to a DNA–DNA hybridization (DDH) value of 70%, and can be used as a threshold for species delineation (Konstantinidis and Tiedje, 2005; Kim et al., 2014).

### Gene synteny analysis

The progressiveMauve option from the Mauve package (Darling et al., 2004) was used with default parameters to perform orthology comparisons and to evaluate gene synteny among the genomes of *F. prausnitzii*. This genome comparison method also predicts syntenic blocks, which reveal the rearrangement events among the genomes (Darling et al., 2004). This analysis was performed using four different datasets: first, using all 17 genomes, and then using the genome subsets of each of the three groups that resulted from ANI analysis.

### Pangenome calculation

The software program OrthoMCL (Li et al., 2003) was used first to define the cluster of orthologous genes and then, the commonly shared and species-specific genes of all the strains and subgroups. The amino-acid sequences from all coding DNA sequences (CDSs) in each genome were first used in an all-vs.-all BLASTp analysis with an *e*-value of 10^−6^; the sequences were then clustered using the MCL algorithm. The CDSs observed in all strains were considered to comprise the core genome, while the CDSs harbored by only one strain were considered to be singleton genes.

To calculate pangenome development, we applied Heap's Law, with the formula *n* = k^*^N^−α^, where *n* is the expected number of genes for a given number of genomes, *N* is the number of genomes, and the other terms are constants defined to fit the specific curve. According to Heap's law, a value of α ≤ 1 is representative of an open pangenome; this means that each added genome will contribute some new genes and the pangenome will increase. Instead, an α value >1 represents a closed pangenome, in which the addition of new genomes will not significantly affect the size of the pangenome. The extrapolations of the curves of the core genome and singletons were calculated using the least-squares fit of the exponential regression decay of the mean values, as represented by the formula *n* = k^*^exp[−x/t]+tg(θ), where n is the expected subset of genes for x number of genomes, exp is Euler's number, and the other terms are constants defined to fit the specific curve. The formula used to calculate the extrapolated curves of core and singleton genes can be used to predict the final number of CDSs if we consider a high number of genomes. In this formula, the value of tg(θ) represents the convergence value of the size of the core genome or the number of new genes (singletons).

## Results

### General features

The number of contigs in the draft genomes varied from 22 to 244. The genome lengths varied by 613,994 bp in size. The GC content varied from 54.81% (*F. prausnitzii* SL3/3) to 58.11% (*F. prausnitzii* CNCM_4541) and the number of predicted CDSs varied from 2,776 to 3,611 (Table 1).

### Phylogeny

**Figure 1:**
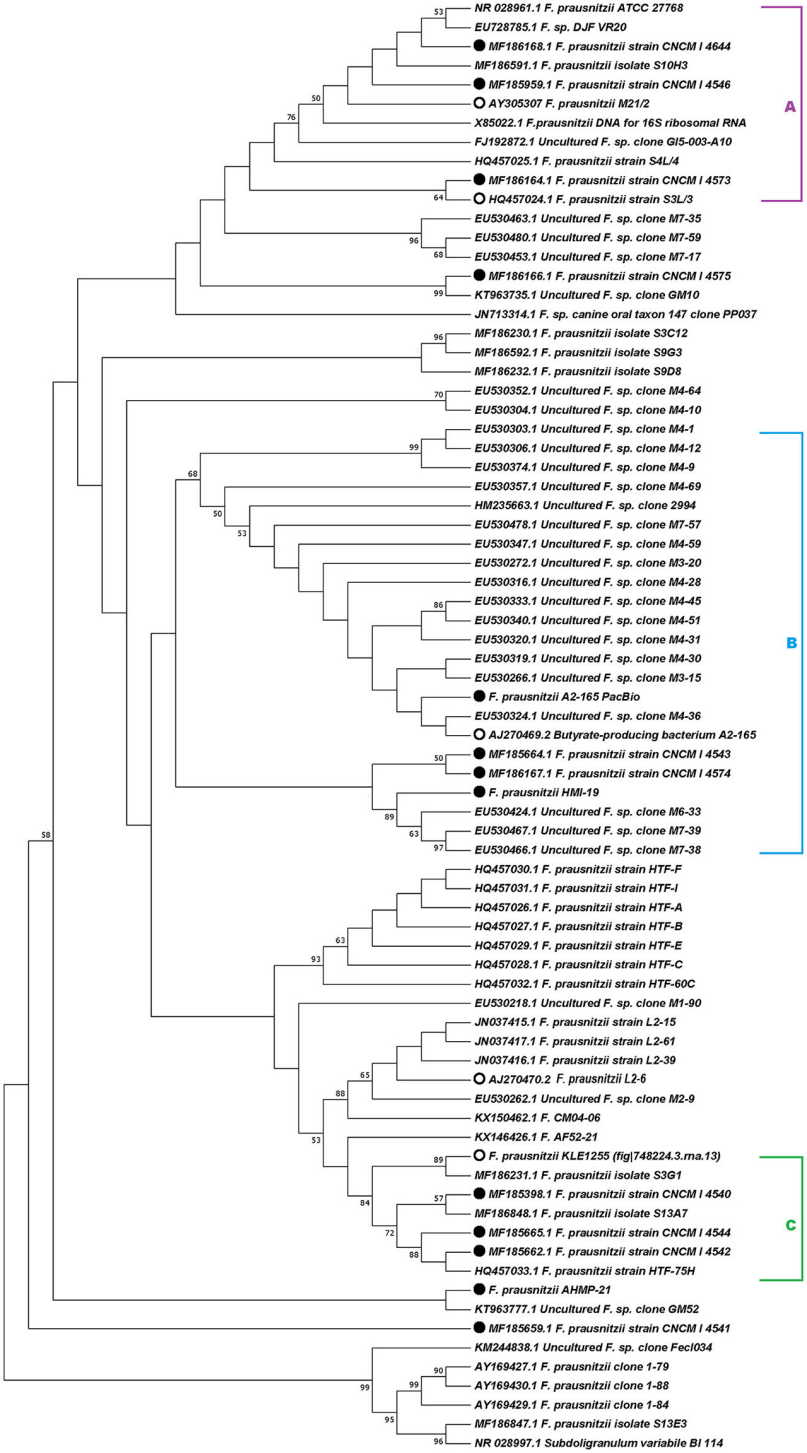
Phylogenetic analysis based on 16S rRNA gene sequences. Evolutionary history was inferred using the maximum-likelihood method based on the Kimura 2-parameter model (Kimura, 1980). The topology of the tree with the highest log likelihood (−3,562.92) is shown. The percentage of trees in which the associated taxa clustered together is shown next to the branches. A discrete Gamma distribution was used to model evolutionary rate differences among sites [5 categories (+G, parameter = 0.1122)]. The tree is drawn to scale, with branch lengths measured as the number of substitutions per site. The analysis involved 76 nucleotide sequences. All positions containing gaps and missing data were eliminated. The bootstrap analysis was performed with 1,000 replicates. Evolutionary analyses were conducted in MEGA7 (Kumar et al., 2016). Accession numbers of 16S rRNA sequences are given in parentheses. Filled circles indicate the strains newly sequenced for this study and open circles indicate the strains retrieved from PATRIC for genomic analysis.

The phylogenetic analysis of 16S rRNA sequences revealed that the genospecies of *Faecalibacterium* can be clustered into different groups. Specifically, the 16S rRNA gene sequences from the new French genomes grouped into clusters A, B, and C (Figure 1), as previously proposed by Martín et al. (2017). The 16S rRNA gene sequence from one Australian isolate, *F. prausnitzii* HMI-19, clustered with sequences in group B, whereas the other Australian sequence (*F. prausnitzii* AHMP-21) did not cluster in any of the proposed groups. Likewise, the 16S rRNA sequences from three other strains—*F. prausnitzii*_CNCM_I-4541, *F. prausnitzii*_CNCM_I-4575, and *F. prausnitzii*_L2-6—did not cluster into any of the three groups proposed here.

**Figure 2:**
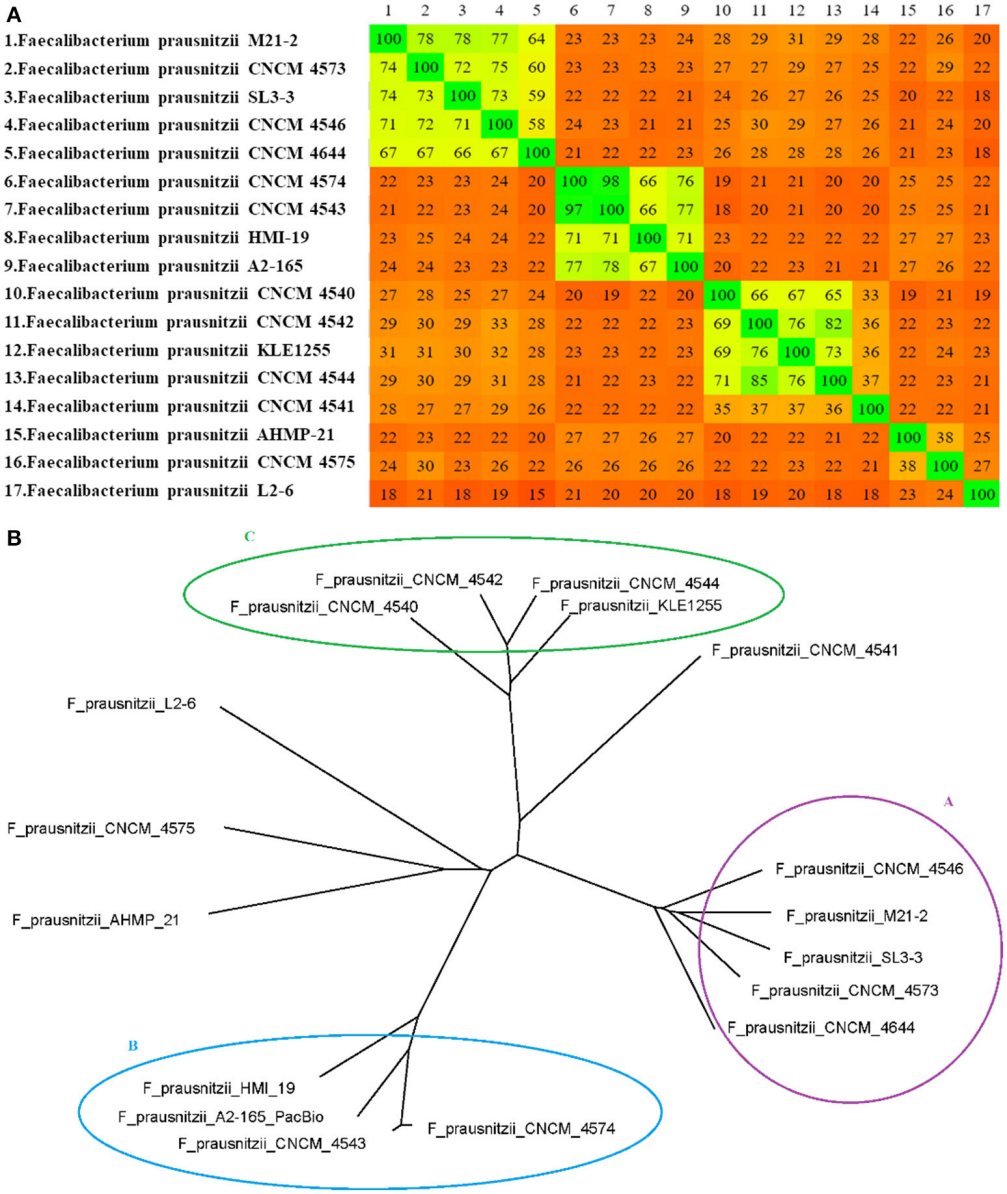
**(A)** Heatmap and **(B)** distance-matrix-based phylogenetic network of *F. prausnitzii*. The numbers in the heatmap show the percentage of similarity between genomes; the colors vary from red (low similarity) to green (high similarity). The network was constructed using SplitsTree software with NeighborNet and equal angle methods, based on a distance matrix from Gegenees software.

The distance matrix generated using Gegenees software was plotted as a heatmap (Figure 2), in which the similarity among genomes varied from ~15% (between *F. prausnitzii*_L2-6 and *F. prausnitzii*_CNCM_I_4644) to ~98% (between the genomes of *F. prausnitzii*_CNCM_I_4543 and *F. prausnitzii*_CNCM_I_4574, which were isolated from the same volunteer). In this analysis, the genomes of *F. prausnitzii*_CNCM_I-4573 and *F. prausnitzii*_SL3/3 clustered together with group A, whereas the genome of *F. prausnitzii*_CNCM_I_4541 was only distantly related to the other strains from group C. As we found with the 16S rRNA analysis, the genome sequences of *F. prausnitzii* AHMP-21, *F. prausnitzii*_CNCM_I-4575, and *F. prausnitzii*_L2-6 did not cluster with any other sequence.

**Figure 3:**
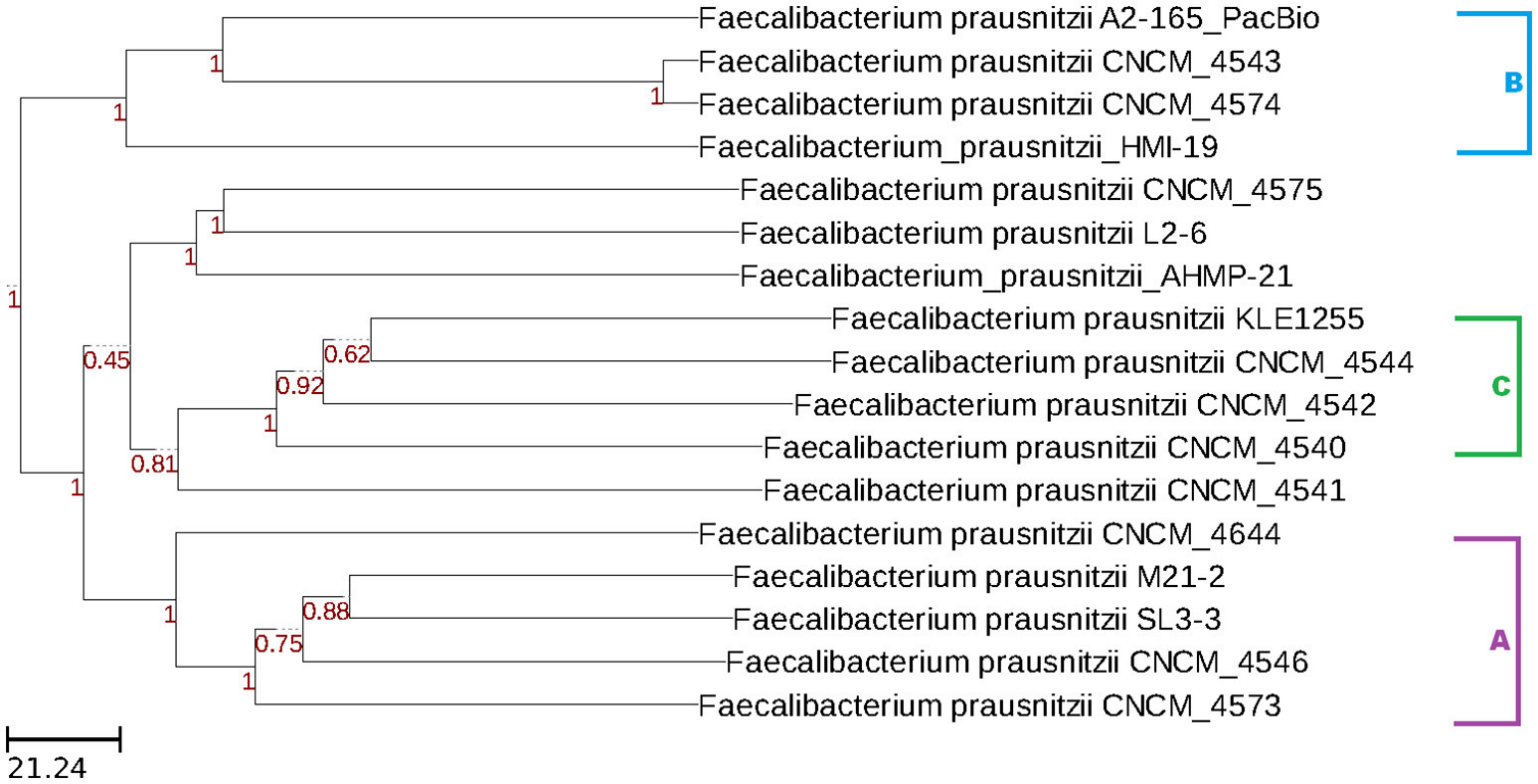
Dendrogram constructed with wgMLST profiles for 17 *F. prausnitzii* genomes. The PGAdb profile from the genomes was used to construct a wgMLST tree using the *Build_wgMLSTtree* module (Liu et al., 2016). Bootstrap values are shown next to the nodes. The dendrogram was constructed with the UPGMA clustering algorithm.

The wgMLST analysis also revealed the presence of three clusters of genomes. Once again, strain *F. prausnitzii*_CNCM_I_4541 was distantly related to other strains in group C, whereas strains *F. prausnitzii* AHMP-21, *F. prausnitzii*_CNCM_I-4575, and *F. prausnitzii*_L2-6 grouped separately from other strains (Figure 3).

### Average nucleotide identity

We performed an Average Nucleotide Identity (ANI) analysis using whole-genome sequence data (Table 2). Using an identity cut-off of 94%, this analysis also revealed the presence of the three clusters revealed by the phylogenetic analyses. In addition, the results of the ANI analysis corroborated those of the phylogenomic and wgMLST approaches in finding that the genome sequences of *F. prausnitzii* AHMP-21, *F. prausnitzii*_CNCM_I-4575, *F. prausnitzii*_CNCM_I-4541, and *F. prausnitzii*_L2-6 did not cluster with any other genome sequence. As estimates of ANI are considered to be the gold standard for bacterial species determination, we used the three groups defined here for all further analyses.

**Table 2:** Average nucleotide identity.

**Strains**	**CNCM_I_4546**	**CNCM_I_4573**	**CNCM_I_4644**	**M21-2**	**SL3-3**	**A2-165_PacBio**	**CNCM_I_4543**	**CNCM_I_4574**	**HMI-19**	**CNCM_I_4540**	**CNCM_I_4542**	**CNCM_I_4544**	**KLE1255**	**AHMP-21**	**CNCM_I_4541**	**CNCM_I_4575**	**L2-6**
CNCM_I_4546	100	97.37	95.03	97.33	97.36	86.23	86.76	86.85	86.02	86.47	87.6	86.88	87.44	85.48	86.57	86.81	85.72
CNCM_I_4573	97.37	100	95.02	97.13	97.19	86.79	86.2	85.99	86.66	87.11	86.86	86.88	87.21	86.01	86.24	88.42	84.64
CNCM_I_4644	95.03	95.02	100	95.09	94.99	86.28	85.88	85.81	85.6	85.81	86.44	86.34	86.57	85.62	85.91	86.48	84.87
M21-2	97.33	97.13	95.09	100	97.36	86.75	86.03	85.9	85.78	86.48	86.8	86.92	87.35	85.58	86.52	86.79	85.43
SL3-3	97.36	97.19	94.99	97.36	100	86.16	86.47	86.44	86.21	86.03	86.67	86.85	87.05	85.6	86.3	86.33	85.34
A2-165_PacBio	86.23	86.79	86.28	86.75	86.16	100	98.08	97.99	97.12	85.21	86.11	85.47	86.22	86.1	85.28	86.32	86.09
CNCM_I_4543	86.76	86.2	85.88	86.03	86.47	98.08	100	99.9	97.08	84.82	85.58	85.42	85.75	85.85	85.2	85.9	85.71
CNCM_I_4574	86.85	85.99	85.81	85.9	86.44	97.99	99.9	100	97.1	84.77	85.71	85.26	85.63	86	85.02	85.85	85.99
HMI-19	86.02	86.66	85.6	85.78	86.21	97.12	97.08	97.1	100	86.08	85.56	85.59	85.59	85.96	85.47	86.1	85.93
CNCM_I_4540	86.47	87.11	85.81	86.48	86.03	85.27	84.82	84.77	86.08	100	97.62	97.57	97.52	85.03	87.65	85.7	85.61
CNCM_I_4542	87.6	86.86	86.44	86.6	86.67	86.11	85.58	85.71	85.56	97.62	100	98.46	98.1	85.62	88.09	85.97	86.13
CNCM_I_4544	86.88	86.88	86.34	86.92	86.85	85.47	85.42	85.26	85.59	97.57	98.46	100	98.14	85.63	88.05	85.87	86.03
KLE1255	87.44	87.21	86.57	87.35	87.05	86.22	85.75	85.63	85.59	97.52	98.1	98.14	100	85.73	87.94	86.52	86.47
AHMP-21	85.48	86.01	85.61	85.58	85.6	86.1	85.85	86	85.96	85.03	85.62	85.64	85.73	100	85.14	88.31	86.21
CNCM_I_4541	86.57	86.24	85.91	86.52	86.3	85.25	85.2	85.02	85.47	87.65	88.09	88.05	87.94	85.14	100	85.25	85.22
CNCM_I_4575	86.81	88.42	86.48	86.79	86.33	86.32	85.9	85.85	86.1	85.7	85.97	85.87	86.52	88.31	85.25	100	86.87
L2-6	85.72	84.64	84.87	85.43	85.34	86.09	85.71	85.99	85.93	85.61	86.13	86.03	86.47	86.21	85.22	86.87	100

*The colors purple, blue, and green corresponds to the clusters A, B, and C respectively*.

### Gene synteny analysis

**Figure 4:**
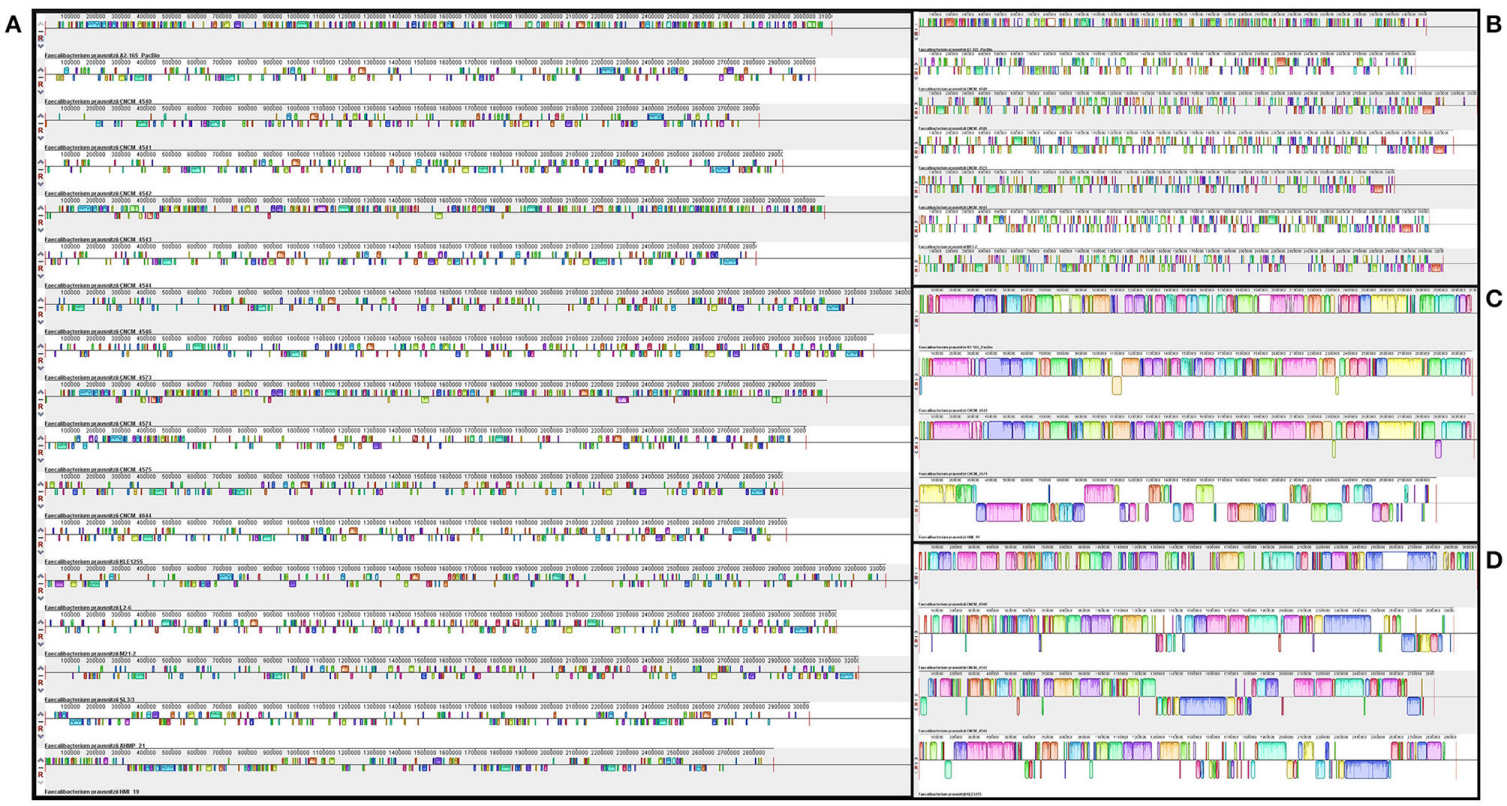
Genomic synteny and gene conservation among the genomes of *F. prausnitzii*. The left side of the figure **(A)** shows the LCBs of all genomes studied here. The right side depicts the LCBs of the genomes within each of the three clusters previously obtained from ANI analysis: top right **(B)**—cluster A, middle right **(C)**—cluster B, and bottom right **(D)**—cluster C.

Mauve software was used to order the contigs within the genomes and to identify and align regions of local collinearity (called Locally Collinear Blocks, or LCBs), which are regions without local rearrangement of probable homologous sequences that are shared by two or more genomes (Darling et al., 2004). In Figure 4, the prediction of LCBs in all strains showed small and numerous regions of homology. When the analysis considered only the genomes within a cluster, the LCBs were larger and less numerous.

### Pangenome calculation

**Figure 5:**
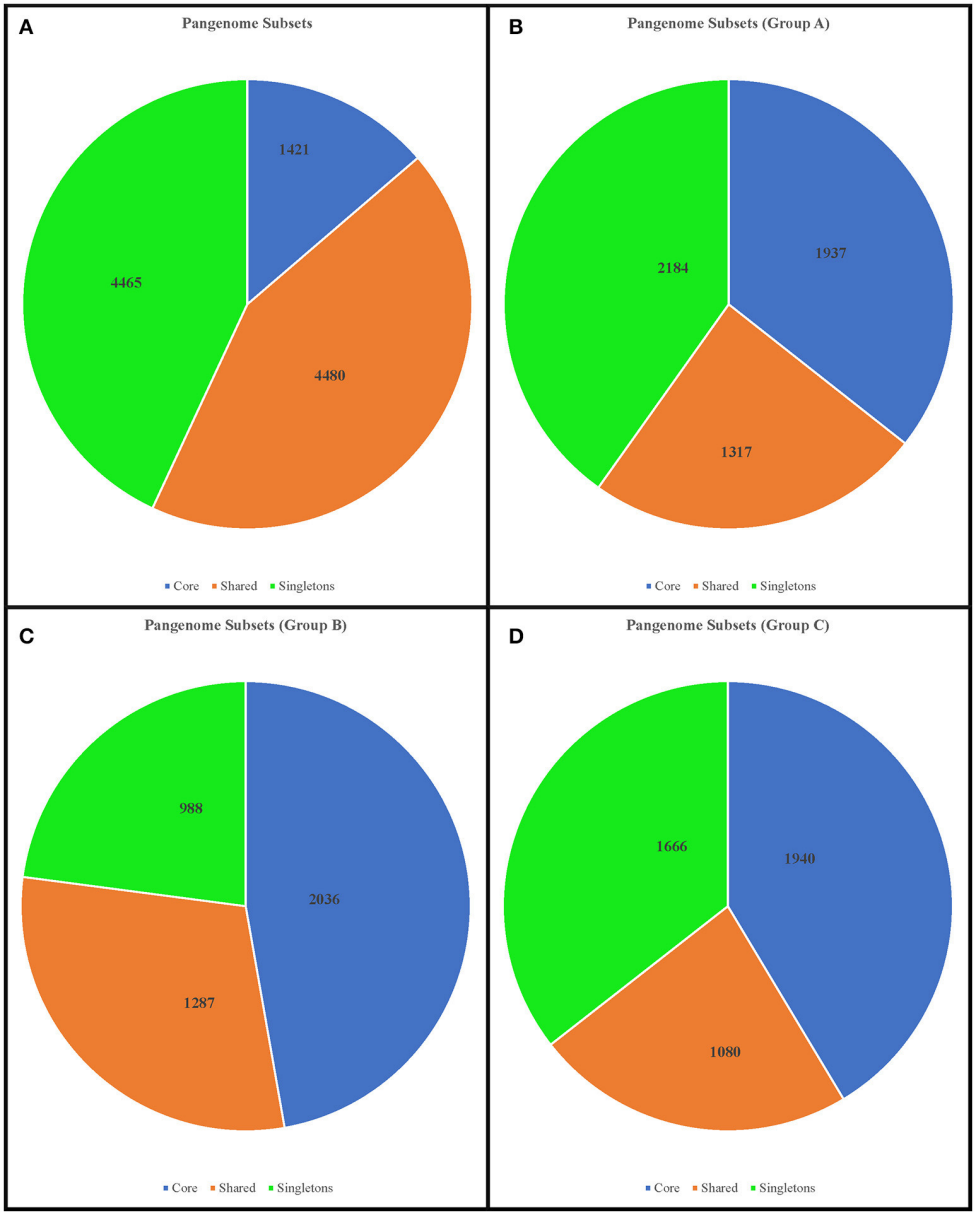
Diagram depicting the subsets of the *Faecalibacterium* pangenome. The numbers represent the coding sequences belonging to each subset. Upper left chart **(A)**: pangenome subsets from an analysis based on all 17 genomes of *Faecalibacterium*. Upper right chart **(B)**: subset based on analysis of 5 genomes from group A. Lower left chart **(C)**: subset based on analysis of 4 genomes from group B. Lower right chart **(D)**: subset based on analysis of 4 genomes from group C.

**Figure 6:**
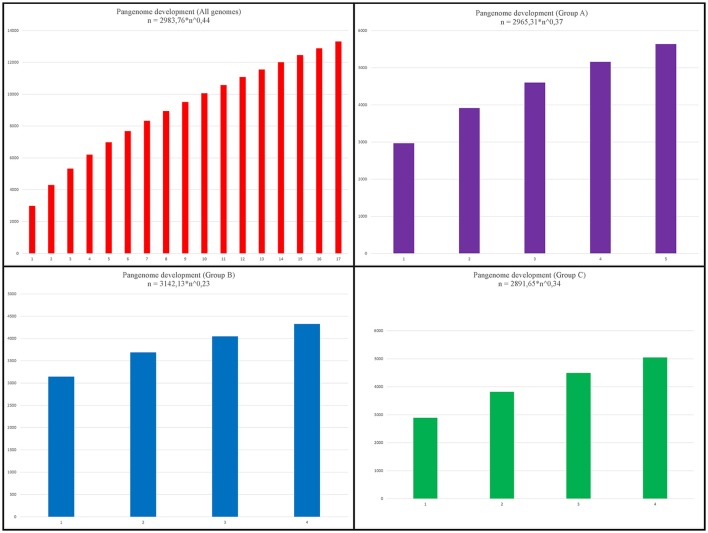
Pangenome development. Upper left chart: pangenome development based on permutations of all 17 genomes of *Faecalibacterium*. Upper right chart: development based on permutations of 5 genomes from group A. Lower left chart: development based on permutations of 4 genomes from group B. Lower right chart: development based on permutations of 4 genomes from group C.

To take a global view of the genome of *Faecalibacterium* and to further explore the genome diversity of this genus, we calculated the size of the pangenome (i.e., the total number of non-redundant CDSs) based on different datasets. When we examined all genomes together, the orthology analysis showed that the pangenome contained a total of 10,366 CDSs (Figure 5A), which corresponded to ~3.33-fold the average total number of genes in each of the 17 strains (3,110.29 CDSs). When we considered only the genomes in group A, we found a total of 5,438 CDSs (Figure 5B), ~1.71-fold the average total number of CDSs in each member strain (3,187.4). The pangenome of group B had 4,311 CDSs (Figure 5C), which was ~1.36-fold the average total number of genes in each member strain (3,159), and group C had 4,686 CDSs in its pangenome (Figure 5D), which was ~1.57-fold the average total number of genes in each member strain (2,991.75). Using the formula α = 1−γ, we inferred that the α value of the pangenome of all genomes was 0.56, indicating that the pangenome is probably open and increasing. Similarly, the extrapolation of the pangenome size calculated for groups A, B, and C generated α values of 0.63, 0.77, and 0.66, respectively (Figure 6).

**Figure 7:**
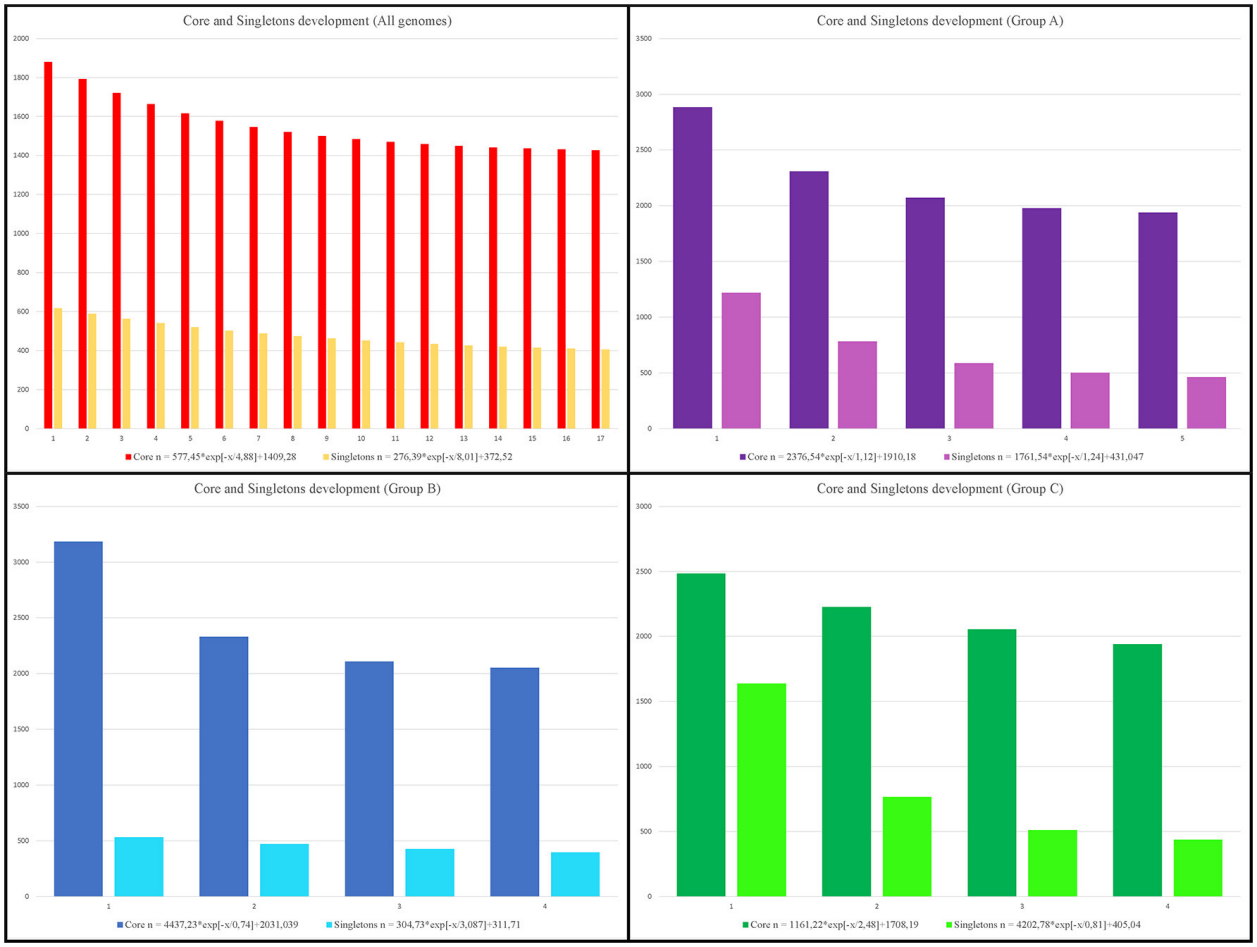
Development of core genome and singletons. Upper left chart: core-genome and singleton development based on permutations of all 17 genomes of *Faecalibacterium*. Upper right chart: development based on permutations of 5 genomes from group A. Lower left chart: development based on permutations of 4 genomes from group B. Lower right chart: development based on permutations of 4 genomes from group C.

Examination of the core genome showed that 1,421 CDSs were shared by all genomes, which corresponded to less than 50% of the average gene content in each genome (3,110.29 CDSs) and represented ~13.71% of the total pangenome. A separate analysis of the core genome of each group revealed 1,937, 2,036, and 1,940 CDSs, respectively, in groups A, B, and C. The subset of CDSs in all genomes considered to be singletons (i.e. unique to a single genome) contained 4,465 CDSs, while within-group analyses revealed 2,184, 988, and 1,666 singleton CDSs, respectively, within groups A, B, and C (Figure 5). By examining the extrapolated curve of the core genome of *Faecalibacterium* ssp., we found that the size of the core genome tended to converge at 1,409 genes, which represented only 13.59% of the pangenome. Within groups A, B, and C, this value increased to 1,910, 2,031, and 1,708 genes, respectively, which represented 35.12, 47.11, and 36.45% of the respective pangenome (Figure 7).

## Discussion

In bacteria, 16S rRNA sequences have been widely used to study phylogenetic relationships. However, this approach is hampered by the fact that several forces that shape the evolution of bacterial genomes act with different strengths on different parts of the genome and on different bacterial lineages (Janda and Abbott, 2007; Chan et al., 2012). Therefore, to determine the diversity within a bacterial genus or species, it is important to consider not only 16S rRNA sequences, but also the whole genome. Despite this, study of the evolutionary history of genus *Faecalibacterium* has largely been conducted through analyses of 16S rRNA sequences. For example, the first study of 16S rRNA gene sequences of *F. prausnitzii* revealed that this species had been misclassified into genus *Fusobacterium* (Wang et al., 1996; Duncan et al., 2002). After that, Lopez-Siles et al. (2012) used this sequence region to propose the existence of two phylogroups within what is currently considered *F. prausnitzii*. Furthermore, a recent study based on 16S rRNA data showed that there are significant differences among the strains of *F. prausnitzii* in resistance to antibiotics and metabolic activities (Martín et al., 2017). Here we compared the 16S rRNA gene sequences of new *F. prausnitzii* isolates to those previously available, and overall, our results challenge the current concept of a division of isolates into two broad phylogroups. As was initially proposed by Martín et al. (2017), the 16S rRNA gene sequences of our new French *F. prausnitzii* isolates can be clustered into three groups (although one, group B, was indeed supported with a lower bootstrap value than the other two). The Australian isolate *F. prausnitzii*_HMI-19 also clustered into group B, while the other Australian isolate, *F. prausnitzii*_AHMP-21, does not cluster within any of the groups proposed at present. Taken together, our analyses would suggest that there is more phylogenetic complexity in the classification of this species than has been previously shown in other studies (Wang et al., 1996; Duncan et al., 2002; Lopez-Siles et al., 2012; Martín et al., 2017). This ambiguity motivated our use of techniques other than 16S analysis in order to better understand the diversity inside genus *Faecalibacterium*.

A whole-genome comparative analysis further validated our findings from the 16S rRNA gene phylogeny. A whole-genome similarity matrix was obtained with Gegenees software and used for a phylogenomic analysis; the resulting phylogenetic tree agreed with the previously performed 16S rRNA analysis in identifying the same three groups of strains: clusters A, B, and C. In this analysis, the genospecies *F. prausnitzii*_CNCM_I_4541 clustered within group C, but the relationship between this strain and other members of group C is very distant, reflecting the low degree of genomic similarity between the former and the latter (~36% similarity). The same pattern was found for *F. prausnitzii* AHMP-21, *F. prausnitzii*_CNCM_I_4575, and *F. prausnitzii*_L2-6, which were grouped together, but at similarity values ranging from ~23 to ~38% (as observed in the heatmap). It is interesting to note that certain strains that were isolated from the same volunteer were quite dissimilar (~27% similarity between FPR_CNCM_I_4573 and FPR_CNCM_I_4575; ~37% between FPR_CNCM_I_4541 and FPR_CNCM_I_4542), suggesting that the same individual may harbor different genospecies. The overall abundance of a given genospecies of *Faecalibacterium* within an individual host may be extremely relevant to the study of human diseases, as this overall abundance depends on the disease under study (Hippe et al., 2016; Lopez-Siles et al., 2016). For example, as part of a case-control study of atopic dermatitis (AD) in Korean subjects, Song et al. (2016) reported that 16S rRNA PCR amplicons from stool samples of AD patients were enriched in those similar to strain L2-6 with respect to other strains of *F. prausnitzii*; they also proposed that strain L2-6 can be differentiated from other strains by the existence of a polycistronic region encoding GalNac uptake and metabolism (Song et al., 2016). Our analysis here showed that this strain does indeed demonstrate a very distinct phylogenetic pattern, which increases its potential for use as a reference strain in future AD studies.

To improve the resolution of our phylogenetic analysis, we also applied a strategy based on wgMLST analysis. As opposed to conventional MLST analysis, which uses only a few housekeeping genes, the wgMLST approach takes advantage of a larger number of tracked loci, enabling higher resolution in intraspecies differentiation (Maiden et al., 2013). Our analysis considered only genes that shared more than 80% coverage and 90% identity. Here, the same three groups of genospecies (A, B, and C) were also detected, and again strain *F. prausnitzii*_CNCM_I_4541 was only distantly related to other members of group C. A group containing the isolates *F. prausnitzii*_AHMP-21, *F. prausnitzii*_CNCM_I_4575, and *F. prausnitzii*_L2-6 was also observed. In sum, each of the three phylogenetic analyses we performed suggested the existence of more than one genospecies within the genus *Faecalibacterium*.

To further corroborate the existence of these potential new “species,” we performed an ANI analysis, which confirmed the new relationships identified in the previous analyses. The ANI analysis supported the classification of *F. prausnitzii*_CNCM_I_4541 as a distinct genospecies separate from group C; likewise, the genomes of *F. prausnitzii*_AHMP-21, *F. prausnitzii*_CNCM_I_4575, and *F. prausnitzii*_L2-6 were found to be quite dissimilar from all other genomes considered.

Using our revised clustering of the *F. prausnitzii* genomes, supported by the ANI results, we then investigated genome diversity via gene synteny analysis and calculations of pangenome. The extent of intra-cluster gene synteny was clearly evident in the Mauve alignments. Furthermore, the number and the lengths of the LCBs in the all-genomes dataset were strikingly different from those in the three intra-cluster datasets, which together indicated a higher degree of genome similarity within than among clusters, particularly with regard to group B. Even within a single genospecies, different genomes had a considerable number of regions with inversions and deletions, which may have arisen from horizontal gene transfer events. Again, though, the genomes from group B were more similar to each other than were the genomes of the other groups.

The same four datasets were used to perform calculations of pangenome. As might be expected, the number of core-genome CDSs was greater within each cluster than within the dataset containing all 17 genomes, which is consistent with the idea that the genomes within a cluster are from the same species. Extrapolations of pangenome development also corroborated this assumption. The α value generated from an analysis of all genomes indicated that the genus *Faecalibacterium* has an open pangenome (α = 0.56), as does each of the groups (α = 0.63, α = 0.77, and α = 0.66, respectively). However, the intra-group α values reveal that these latter pangenomes are increasing in size more slowly than the pangenome of all species (as indicated by higher α values). This means that, if we consider all the genomes to be part of the genus *Faecalibacterium*, each new genome sequenced will increase substantially the number of non-redundant genes in this genus. On the other hand, the genomes within each group tend to be more clonal, and newly sequenced genomes included within these groups will have a less prominent effect on the number of non-redundant genes. We likewise arrived at the same conclusion by analyzing the development of the core genome and singletons: the final core genome tended to be larger within each genospecies than within the all-genome analysis. This phylogenetic approach to pangenome analysis revealed patterns that were totally in accordance with the results of our other analyses.

## Concluding remarks

Here, we used a variety of methods to analyze 16S rRNA and whole genome data, which together showed that: (i) the current application of phylogroups to differentiate among strains of *F. prausnitzii* should be revised; (ii) this genus contains at least three separate clusters, spanning both phylogroups I and II, which are all derived from a common recent ancestor; and (iii) some strains (e.g., *F. prausnitzii* AHMP-21*, F. prausnitzii*_L2-6, and *F. prausnitzii*_CNCM_I_4575) appear to represent a deeper, more divergent branch of “*Faecalibacterium prausnitzii*.” Collectively, our results provide evidence for the reconsideration of the phylogenetic and genomic relatedness among strains currently assigned to *F. prausnitzii*. In addition, they highlight the need for lineage (strain-based) differentiation within this genus to better define how specific members might be associated with positive or negative host interactions. Such lineage-specific variations might not only explain the variable abundances of *F. prausnitzii* linked with adverse health outcomes (e.g., atopic dermatitis, Crohn's disease, and ulcerative colitis; Swidsinski et al., 2008; Hansen et al., 2012), but also provide new opportunities for the diagnosis and strain-specific treatment of gut inflammation and associated diseases. Also, to the best of our knowledge, this is the first work to combine an analysis of pangenome development with ANI analysis in order to corroborate the assignment of strains to new species. Here, we propose that pangenome subsets and the α value generated by these analyses may be used as an alternative approach, together with ANI, for the *in silico* classification of new species. Although low α values may be found inside a species cluster, due to a high degree of variation among genomes arising from intense horizontal gene transfer events, a high intra-cluster α value may be considered a good indicator of a new, more-clonal species inside the genus.

## Author contributions

LB, SB, RM, VR, MT, SM, FC, HS, LGB, MM, PL, VA, JC, and SS designed the experiments, revised the manuscript critically, and participated in the design of the project. LB, SB, RM, VR, SM, FC, and SC performed the experiments and analysis. LB and SC drafted the manuscript.

### Conflict of interest statement

The authors declare that the research was conducted in the absence of any commercial or financial relationships that could be construed as a potential conflict of interest.
